# Left ventricular torsional mechanics and myocardial iron load in beta-thalassaemia major: a potential role of titin degradation

**DOI:** 10.1186/1471-2261-14-49

**Published:** 2014-04-12

**Authors:** Mei-pian Chen, Shu-na Li, Wendy WM Lam, Yuen-chi Ho, Shau-yin Ha, Godfrey CF Chan, Yiu-fai Cheung

**Affiliations:** 1 Department of Paediatrics and Adolescent Medicine, Queen Mary Hospital, The University of Hong Kong, Hong Kong, China; 2 Department of Radiology, Queen Mary Hospital, Hong Kong, China; 3 Division of Paediatric Cardiology, Department of Paediatrics and Adolescent Medicine, The University of Hong Kong, Queen Mary Hospital 102, Pokfulam Road, Hong Kong, China

**Keywords:** Thalassaemia, Ventricular rotation, Ventricular mechanics, Exercise echocardiography, Titin

## Abstract

**Background:**

Iron may damage sarcomeric proteins through oxidative stress. We explored the left ventricular (LV) torsional mechanics in patients with beta-thalassaemia major and its relationship to myocardial iron load. Using HL-1 cell and B6D2F1 mouse models, we further determined the impact of iron load on proteolysis of the giant sarcomeric protein titin.

**Methods and results:**

In 44 thalassaemia patients aged 25 ± 7 years and 38 healthy subjects, LV torsion and twisting velocities were determined at rest using speckle tracking echocardiography. Changes in LV torsional parameters during submaximal exercise testing were further assessed in 32 patients and 17 controls. Compared with controls, patients had significantly reduced LV apical rotation, torsion, systolic twisting velocity, and diastolic untwisting velocity. T2^*^ cardiac magnetic resonance findings correlated with resting diastolic untwisting velocity. The increments from baseline and resultant LV torsion and systolic and diastolic untwisting velocities during exercise were significantly lower in patients than controls. Significant correlations existed between LV systolic torsion and diastolic untwisting velocities in patients and controls, both at rest and during exercise. In HL-1 cells and ventricular myocardium of B6D2F1 mice overloaded with iron, the titin-stained pattern of sarcomeric structure became disrupted. Gel electrophoresis of iron-overloaded mouse myocardial tissue further showed significant decrease in the amount of titin isoforms and increase in titin degradation products.

**Conclusions:**

Resting and dynamic LV torsional mechanics is impaired in patients with beta-thalassaemia major. Cell and animal models suggest a potential role of titin degradation in iron overload-induced alteration of LV torsional mechanics.

## Background

Iron overload cardiomyopathy is well documented in patients with beta-thalassaemia major. Nonetheless, the left ventricular (LV) function usually remains relatively normal as assessed by conventional echocardiographic imaging until late in the cardiomyopathic process [1]. These conventional echocardiographic indices are derived primarily at resting condition from linear deformation of LV myocardium [2,3]. Increasing understanding of cardiac mechanics have been gained from evaluation of resting [4] and dynamic [5,6] LV torsional mechanics, which provide information on systolic and diastolic LV performance and insights into myocardial function at a cellular level.

Left ventricular torsional mechanics plays an important role with respect to LV ejection and filling [7,8] and is sensitive changes in regional and global LV function [9,10]. It is characterized by systolic twisting and diastolic untwisting about its long axis as a result of the opposite rotation of the cardiac apex and base. The twisting motion of the left ventricle is important in the Frank-Starling mechanism [11], while the rate of untwisting has been shown to be a relatively load-independent index of diastolic function [12].

Changes at a cellular level may perhaps also be reflected by alterations of LV torsional mechanics. Transplanted human hearts in early rejection have been shown to display alterations in systolic twisting and diastolic untwisting patterns [13]. Remodeling after myocardial infarction in animal models is associated with altered LV torsion [14]. In experimental cardiomyopathy models, alterations of sarcomeric proteins, in particular transmural gradient of titin isoforms [15], has been shown to relate closely to LV torsion. In childhood cancers survivors who had received anthracycline therapy, we have shown abnormalities of LV torsional mechanics that precede changes in LV global function [16]. Importantly, anthracycline is known to induce titin protelolysis [17]. Accumulating evidence suggests oxidative stress may induce degradation of titin via the activity of calpain-1 [17] or matrix metalloproteinase-2 [18] or both. In iron overloading situations, excessive free iron within the cardiomyocytes that causes oxidative damage [19,20] may potentially degrade titin and result in early alteration of LV torsional mechanics.

In this prospective study, we explored the resting and dynamic LV torsional mechanics in patients with beta-thalassaemia major and its relationship with myocardial iron load. Given the initial echocardiographic findings of impaired LV torsion, systolic twisting and diastolic untwisting velocities in patients compared with healthy subjects, we further determined using HL-1 cell and mouse models of iron overload the impact of iron load on titin proteolysis.

## Methods

### Subjects

Forty-four patients with beta-thalassaemia major and normal LV shortening fraction as assessed by two-dimensional echocardiography were recruited. Patients with diabetes mellitus and thyroid dysfunction were excluded. The chelation therapy of patients was noted. Thirty-eight healthy subjects were recruited as controls. These included healthy siblings of thalassaemia patients and voluntary staff members and their friends and relatives. The Institutional Review Board of the University of Hong Kong/Hospital Authority Hong Kong West Cluster approved the study and all of the patients gave informed written consent.

All subjects rest for at least 15 minutes before blood pressure and cardiovascular assessments. The body weight and height were measured and the body mass index and body surface area calculated accordingly. For thalassaemic patients, the study was performed within one week of blood transfusion to minimize potential confounding influence of anaemia on the assessment results.

### Echocardiographic assessment

Echocardiographic assessments were performed using the Vivid 7 ultrasound machine (GE Medical System, Horten, Norway). All of the following echocardiographic data were obtained at rest. The parameters of torsional mechanics were obtained both at rest and during submaximal exercise. The average values of echocardiographic indices from three cardiac cycles were used for analyses.

Left ventricular torsion was assessed by two-dimensional speckle tracking echocardiography as reported previously [16]. Briefly, the LV apical and basal short-axis planes were acquired in consecutive fashion at a frame rate of 60-80/second. The basal level was defined as the level of the mitral valve, while the apical one was defined as the level of LV cavity alone with no visible papillary muscles. Using customized EchoPAC software (GE Medical Systems), a speckle tracking region of interest was generated for evaluation of global LV rotation at the basal and apical levels and the resultant LV peak torsion, systolic twisting velocity, and diastolic untwisting velocity.

### Exercise testing

Submaximal exercise testing was performed with a supine bicycle according to the McMaster cycle ergometer protocol [21]. The initial workload was 25 W with a 25 W increase in resistance at 2-minute intervals. Echocardiographic acquisitions of the basal and apical short-axis plane rotation were performed when the heart rate reached 70% of the age-predicted peak heart rate.

### MRI assessment of iron load in patients

T2^*^ cardiac magnetic resonance (CMR) was performed as described previously [22] and in detail in Additional file 1 in 31 of the 44 patients who agreed to the study.

### Iron loading of HL-1 cardiomyocytes

HL-1 cardiomyocytes were kindly provided by Professor W.C. Claycomb (Louisiana State University Health Science Center, New Orleans, LA, USA) [23]. These cells are currently the only available cardiomyocte cell line that divides continuously while maintaining a differentiated cardiac phenotype. The HL-1 cells were cultured in accordance with the instructions provided and details are described in Additional file 1. For iron loading, the cells were exposed to 600 μM of FeCl_3_ for 72 hours.

### Mouse model of iron load

Iron-loaded mouse model was generated as described previously [24]. Seven-week-old male B6D2F1 mice were purchased from The Jackson Laboratory (Bar Harbor, Maine, USA). This part of the study was approved by the Department of Health, Hong Kong SAR, and the Committee on the Use of Live Animals in Teaching and Research, The University of Hong Kong. The investigations conform with the Guide for the Care and Use of Laboratory Animals published by the US National Institutes of Health and the ethical policy of our institution is listed as being compliant with that of NIH (assurance number A5773-01). Mice were acclimatized for at least one week before experiments. The 8-week-old male B6D2F1 mice were given intra-peritoneal injection of iron dextran (50 mg iron/ml; Sigma, St Louis, MO, USA), at 3.1 mg per 25 g body wt per day on a 5 days/week schedule, for 13 weeks. Control mice received 10% dextrose (Sigma, St Louis, MO, USA). Mice were sacrificed by an overdose of isoflurane anaesthesia until lack of respiration for 5 minutes to ensure euthanasia, followed by cervical dislocation. Ventricular tissues were stored at -80°C until analysis.

### Immunocytochemistry, immunohistochemistry and confocal microscopy

HL-1 cells were fixed and permeated with Cytofix/Cytoperm™ solution (Becton Dickinson, Franklin Lakes, NJ, USA), followed by washes with Perm/Wash™ buffer (Becton Dickinson, Franklin Lakes, NJ, USA). Non-specific antibody binding was blocked by incubation with 5% bovine serum albumin (BSA). Titin PEVK domain was stained with primary antibody 9D10 (Developmental Studies Hybridoma Bank at the University of Iowa, Iowa, USA) 1:100 diluted in 5% BSA, followed by secondary antibody Cy™5 (Invitrogen, Life Technologies, Grand Island, NY, USA) 1:100 diluted in 5% BSA. Nuclei were counterstained with DAPI. The patterns of titin staining were analyzed by Zeiss LSM 510 Confocal Microscope (Zeiss, Göttingen, Germany).

For determination of titin pattern in mouse heart tissue, 7 μm-thick cryosection slides of heart tissue were fixed and permeated with 0.2% PBST, and then blocked with 10% normal rabbit serum (NRS) in 0.2% PBST. Titin was stained with antibody 9D10 in 5% NRS and subsequently with the secondary antibody same as aforementioned. Confocal images were analyzed by software ImageJ (NIH, Bethesda, Maryland, USA).

### Gel electrophoresis for titin analysis

Titin was analyzed in ventricular extracts (iron-loaded, n = 4; control, n = 4) using gel electrophoresis as described previously^25^ with details provided in Additional file 1.

### Statistical analysis

Clinical data are presented as mean ± SD. The demographic and echocardiographic parameters of patients were compared with those of control subjects using unpaired Student's *t* test. The resting and exercise echocardiographic indices were compared by paired Student’s *t* test, while the magnitude of changes in torsional indices from the resting to exercise state in patients and controls was compared by unpaired Student’s *t* test. Relationships between systolic torsion and diastolic untwisting velocity at rest and exercise of the entire cohort and within patient and control groups, and associations between torsional indices and iron load were assessed by Pearson correlation analysis. In-vitro study data are presented as mean ± SEM and variables between two groups were compared by unpaired Student’s *t* test. Statistical analyses were performed using SPSS version 16 (SPSS, Inc., Chicago, Illinois) and GraphPad Instat 3 (GraphPad Software, Inc., San Diego, CA, USA). A p < 0.05 was regarded as statistically significant.

## Results

### Subjects

Forty-four patients (19 males) aged 25 ± 7 years were recruited. Chelation therapy regimen at the time of study were deferiprone with deferoxamine in 26 patients, deferasirox in 9, deferoxamine in 7, and deferiprone in 2. Based on T2^*^ cardiac magnetic resonance performed in 31 patients, 8 patients had evidence of myocardial iron overload (T2^*^ ≤ 20 ms). All of the patients were free from cardiac symptoms. Thirty-eight (19 males) healthy young adults aged 23 ± 6 years (p = 0.10) were recruited as controls. Patients compared with controls were lighter (47.5 ± 9.2 kg vs 58.3 ± 11.8 kg, p < 0.001) and had a smaller body surface area (1.45 ± 0.17 m^2^ vs 1.64 ± 0.18 m^2^, p < 0.001).

### Resting LV torsional mechanics

At baseline, patients had significantly lower LV torsion, systolic twisting velocity, and diastolic untwisting velocity compared with controls (all p > 0.05) (Table 1). The significantly lower LV torsion was related to the reduced apical but not basal rotation. Even in patients with T2^*^values >20 ms, the LV apical rotation, torsion, and systolic twisting velocities were significantly lower than those of controls. While these parameters were also lower in the 8 patients with myocardial iron overload (T2^*^ ≤ 20 ms), the small number limits the power to detect statistical significant differences in these variables except for apical rotation. T2^*^ findings correlated inversely with LV diastolic untwisting velocity (r = -0.46, p = 0.013), but not with peak systolic torsion (p = 0.54) twisting velocity (p = 0.65). Hence, the greater the myocardial iron load, the lower the diastolic untwisting velocity.

**Table 1:** Comparison of torsional parameters at rest between patients and controls

	**All patients (n = 44)**	**Patients with CMR performed**		**Controls (n = 38)**
		T2* > 20 ms (n = 23)	T2* ≤ 20 ms (n = 8)	
Basal rotation (degree)	−4.4 ± 2.0	−4.6 ± 2.1	−4.5 ± 1.7	−3.7 ± 2.8
Apical rotation (degree)	9.3 ± 3.6*	9.3 ± 3.3*	8.5 ± 4.3*	12.5 ± 5.1
LV torsion (degree)	11.8 ± 3.3*	11.7 ± 3.5*	12.1 ± 3.5	13.5 ± 3.1
Systolic twisting velocity (degree/s)	93.7 ± 21.9*	97.6 ± 21.1*	98.9 ± 20.1	115.2 ± 36.9
Diastolic untwisting velocity (degree/s)	−98.2 ± 23.3*	−105.9 ± 21.4	−93.1 ± 25.9	−109.8 ± 26.3

CMR = cardiac magnetic resonance.

^*^p < 0.05 vs controls.

### Dynamic LV torsional mechanics

**Figure 1:**
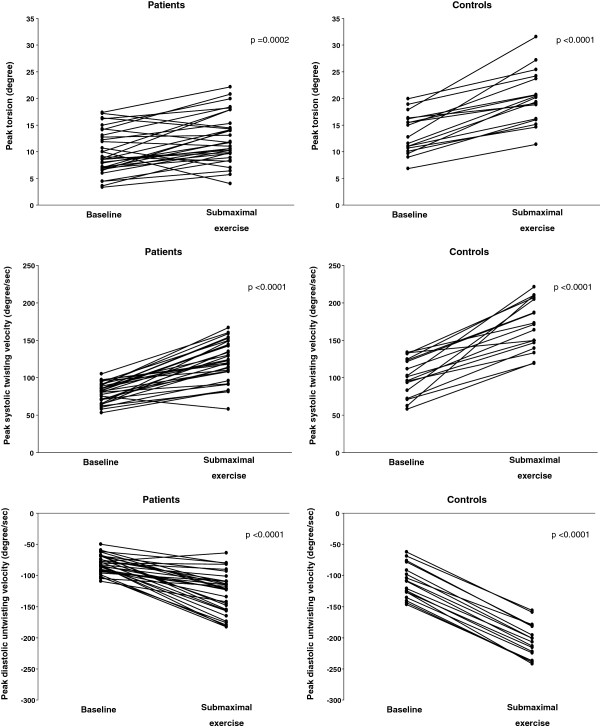
Left ventricular peak torsion, systolic twisting velocity, and diastolic untwisting velocity at baseline and during submaximal supine bicycle exercise in patients and controls.

Thirty two patients and 17 controls agreed to undergo further submaximal exercise testing in the exercise laboratory. During supine bicycle exercise testing, both patients and controls showed significant increase in peak LV torsion and systolic twisting and diastolic untwisting velocities (all p < 0.05) (Figure 1). The magnitude of changes from baseline was, however, significantly smaller in patients than controls for all of the three parameters: peak torsion (2.7 ± 3.6 degree vs 6.9 ± 3.5 degree, p < 0.001), systolic twisting velocity (42.5 ± 22.4 deg/s vs 69.2 ± 33.2 deg/s, p = 0.002), and diastolic untwisting velocity (-46.0 ± 29.8 deg/s vs -95.8 ± 9.5 deg/s, p < 0.001). Hence, when target heart rate was reached during submaximal exercise, patients exhibited significant worse LV torsional mechanics: peak torsion (12.5 ± 4.5 degree vs 20.3 ± 5.0 degree, p < 0.001), systolic twisting velocity (122.4 ± 26.7 deg/s vs 170.2 ± 33.3 deg/s, p < 0.001), and diastolic untwisting velocity (-127.7 ± 33.0 deg/s vs -205.6 ± 27.4 deg/s, p < 0.001). There were no correlations between T2^*^findings and the various LV torsional parameters during submaximal exercise (all p > 0.05).

### Relationships between systolic torsion and diastolic untwisting

**Figure 2:**
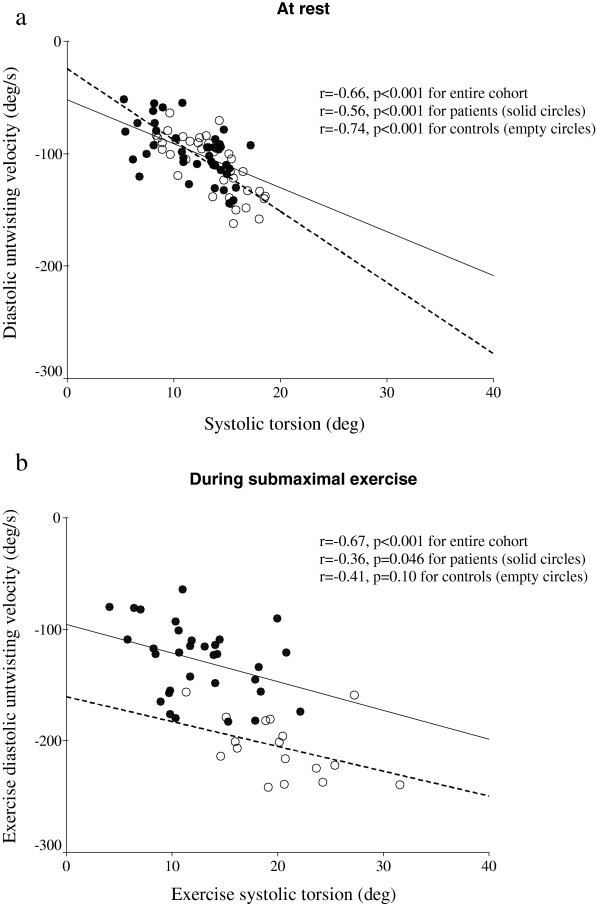
Correlations between systolic torsion and diastolic untwisting velocity (a) at rest, and (b) during submaximal exercise in patients (solid circles) and controls (empty circles).

At rest, significant negative correlations existed between LV systolic torsion and diastolic untwisting velocities in both patients and controls (Figure 2a). Similar relationships existed during submaximal exercise, albeit with smaller correlation coefficients with subgroup analyses (Figure 2b).

### Disruption of titin pattern in HL-1 cells and iron-loaded mouse model

**Figure 3:**
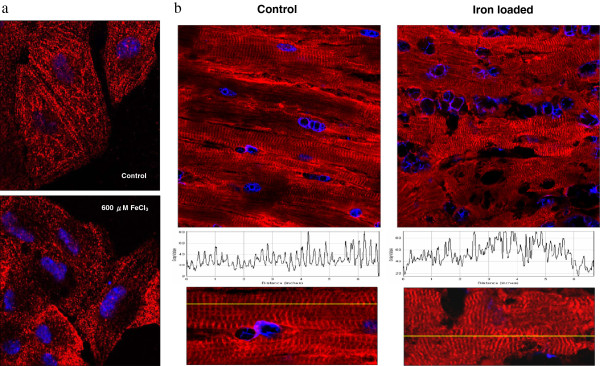
**Confocal microscopic examination of titin disruption in cell culture and myocardial tissue. (a)** Confocal microscopy showing disruption of sarcomeric structure stained for titin in HL-1 cell culture when exposed to iron (III) chloride (FeCl_3_) for 72 hours. **(b)** Confocal microscopy showing disorganized pattern of titin stain with loss of the striated pattern in ventricular myocardial tissue of a mouse overloaded with iron and the normal striations seen in a control mouse (upper panel). The curves in the middle panel represent the fluorescent signal intensity along the yellow lines as shown in the lower panel.

Figures 3a show disruption of the organized sarcomeric structure stained for titin in normal HL-1 cell culture when exposed to FeCl_3_ for 72 hours. After 13 weeks of intra-peritoneal iron dextran administration, the ventricular tissue of B6D2F1 mice showed disorganized pattern of titin stain with loss of the normal striated pattern seen in control heart tissue (Figure 3b).

### Degradation of titin in iron-loaded mouse heart tissue

**Figure 4:**
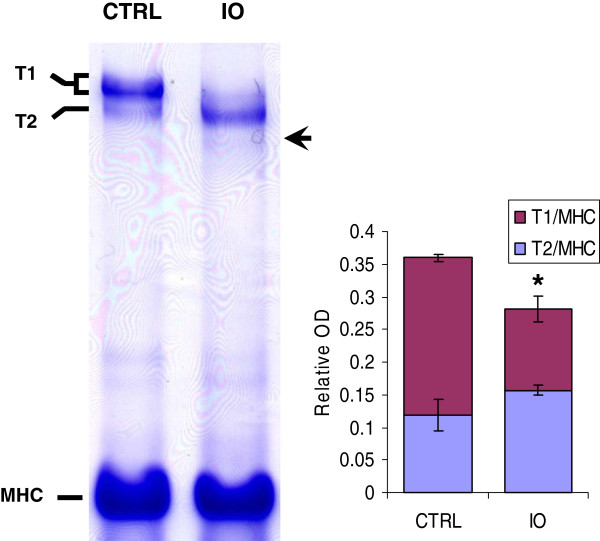
**Gel electrophoresis for analysis of titin degradation in ventricular tissue obtained from control (CTRL) and iron overloaded (IO) mice.** Ventricular myocardium of IO mice showed significant reduction of T1/(myosin heavy chain) MHC and increase in T2/MHC ratios and the appearance of a faint titin degradation subfragment (arrow). (*p < 0.05 vs controls).

Iron-induced titin degradation was quantified by gel electrophoresis (Figure 4). Control heart tissues expressed predominantly T1 bands, which contained titin isoforms N2BA and N2B, and a minor T2 band, the titin degradation product. By contrast, iron-loaded heart tissues showed an increase in T2/(myosin heavy chain) MHC ratio and a decrease in T1/MHC ratio (p < 0.05), and appearance of a faint titin degradation subfragment.

## Discussion

The important findings of the present study are 1) impaired LV torsional mechanics in patients with beta-thalassaemia major at rest, 2) diminished incremental response of systolic twisting and diastolic untwisting parameters during submaximal exercise stress, 3) coupling of systolic torsion and rate of diastolic untwisting, 4) an inverse relationship between myocardial iron load and LV diastolic untwisting velocity, and 5) evidence of titin disruption and degradation in cell and mouse models of iron load, implicating its potential pathogenetic role in causing abnormalities of resting and exercise LV torsional mechanics found in patients clinically.

### Torsional mechanics in thalassaemia

The understanding of torsional mechanics in patients with beta-thalassaemia major has been limited. Using vector velocity imaging, Gareau et al. have recently reported on reduced LV torsion and apical rotation in patients with beta-thalassaemia major and Blackfan-Diamond anaemia with low T2^*^ value (≤20 ms) [25]. Assessments were performed at rest and rates of systolic twisting and diastolic untwisting were not determined. In a relatively small cohort of 19 patients with various causes of anaemia requiring regular blood transfusion, Seldrum et al. found using CMR reduced apical rotation in patients with (T2^*^ ≤ 20 ms) and without (T2^*^ > 20 ms) significant myocardial iron load and decreased LV torsion only in those with T2^*^ ≤ 20 ms compared with controls [26]. Differences in LV twisting and untwisting rates could not be demonstrated, probably due to limited statistical power. Recently, Monte et al. found using speckle tracking echocardiography also reduced apical rotation and LV twist and torsion [27].

While our findings corroborate those reported previously, we found additionally significant reduction of rates of LV systolic twisting and diastolic untwisting in our patient cohort. Albeit modest and without implications on causality, the inverse relationship between iron load and diastolic untwisting velocity may have pathophysiologic significance. Rapid LV diastolic untwisting has been shown to correlate with the time constant of relaxation during isovolumic relaxation [12] and intraventricular pressure gradient after mitral opening, and hence regarded as an important determinant of LV suction [6] and early LV diastolic function [28]. Indeed, impaired LV relaxation as assessed by conventional indices has been shown to precede systolic function in beta-thalassaemia major [29,30] and myocardial T2^*^ values have also been shown to correlate with CMR-derived early to late diastolic ventricular filling [31].

### Exercise torsional mechanics

Using dobutamine stress echocardiography, we [32] and others [33] have shown reduced LV contractile reserve in thalassaemia patients. To our knowledge, this is the first study to assess dynamics of LV torsional mechanics in these patients during exercise stress. Our findings of increased LV torsion and rates of twisting and untwisting in both controls and patients, albeit differing in magnitude, concur with the reported physiological adaptive LV torsional mechanics during exercise in healthy subjects [5,6]. Nonetheless, this adaptive response was significantly blunted in our patients, which may have implications on efficient LV filling during exercise and exercise incapacity. Enhanced LV untwisting at exercise, due to the release of energy stored during LV twist, plays a key role in maintaining LV filling pressure. This acts through an increase in LV suction and intraventricular pressure gradient with shortening of diastole during exercise [6]. Blunting of the augmentation of LV twisting and untwisting response in our patients, and which has also been reported to occur with aging [34] and in heart transplant recipients [35], may potentially lead to diastolic dysfunction and increased diastolic filling pressure during exercise. Based on the present findings, altered LV untwisting should perhaps be considered as a possible mechanism contributing to impaired exercise capacity in thalassaemia major patients [36].

### Systolic-diastolic coupling

Close correlations between systolic torsion and diastolic untwisting velocity in our patients and controls both at rest and during exercise reflect systolic-diastolic coupling, a phenomenon demonstrated previously in normal subjects at rest [6,37] and during exercise [5]. Apart from being an active relaxation process, LV diastolic untwisting is also a consequence of release of stored energy from compressed titin [38], the giant sarcomeric protein, and the elastic component of the myocardial interstitium [39]. There is further evidence to suggest that the sequence of LV torsion followed by rapid untwisting is a manifestation of elastic recoil, linking systolic cardiac contraction to diastolic filling [6]. It is intriguing therefore that during exercise, for similar degree of LV torsion, the diastolic untwisting velocity appeared lower in patients compared with patients (Figure 2), implicating that the diminished force of recoil occurs not only at rest but is further exaggerated during exercise stress in patients. While the exact mechanism of altered torsional mechanics at rest and during exercise in thalassaemia patients remains to be unveiled, given the demonstrated development of restoring force by cardiac titin in shortened cardiomyocytes [38], we further tested using cell and animal models the hypothesis that iron load may induce titin degradation.

### Titin degradation

Titin, the largest protein in the body, acts as a structural, elastic, and signaling molecule in cardiomyocytes [40]. In particular, its role as a bidirectional spring that contributes to passive myocardial stiffness and elastic recoil of cardiomyocytes has been the focus of attention in cardiac diseases. Titin is vulnerable to damage by oxidative stress [41], which is characteristic of iron overload [19,20]. Our findings in cell and animal models of iron load support the notion that iron overload causes disorganization and degradation of cardiac titin. Although direct evidence of titin degradation being linked to alteration of LV twist is lacking based on the current study design, several pieces of evidence suggest that this may probably be the case: i) titin has been shown to develop restoring force in rat cardiomyocytes and a major determinant of diastolic function [38], ii) change of titin isoform induced by ventricular pacing in an animal model of heart failure has been associated with reduced systolic twist and diastolic untwisting [15], and iii) in survivors of childhood cancers who had received anthracyclines, which have been shown to induce titin proteolysis in vitro [17], we [16] and others [42] have demonstrated impaired LV torsional mechanics.

### Clinical implications

Detection in our patient cohort of abnormal LV systolic and diastolic torsional parameters despite normal conventional and tissue Doppler indices of LV function suggests the greater sensitivity of torsional parameters in detecting subtle LV dysfunction in thalassaemia major, although the use of these parameters in accurate quantification of myocardial iron load appears limited. As alluded to earlier, altered LV untwisting may perhaps contribute to impaired exercise capacity documented in thalassaemia major patients [36]. Our demonstration of a potential role of titin degradation in causing altered LV mechanics has potential implications in the development of novel therapeutic strategies for iron overload cardiomyopathy. Calpain has been shown to mediate titin proteolysis with induction by anthracyclines [17], while matrix metalloproteinase-2 has been demonstrated to contribute to titin degradation in myocardial ischaemia-reperfusion injury [18]. While little information is available to date on therapeutic measures to improve LV twist mechanics [43], further mechanistic definition of titin degradation in iron load may shed light on the application of pharmacological inhibition of specific proteases in preventing and treating cardiac dysfunction related to iron overload.

### Limitations

Several limitations to this study warrant comments. Firstly, our patients are relatively young with no congestive heart failure. Whether the findings hold true for those with clinically manifested iron induced cardiomyopathy require further studies for confirmation. Nonetheless, impaired LV torsional mechanics has been well documented in other causes of heart failure [4,10]. Secondly, the small number of patients with myocardial iron overload has limited statistical power to unveil other aspects of abnormal torsional mechanics apart from reduced apical rotation. Thirdly, it would have been ideal to compare resting and dynamic torsional mechanics among patients receiving different chelation therapies, although the relatively small number of patients limits the statistical power to do so in this study. Fourthly, while the present study provides the proof of concept that iron overload induces titin degradation, the mechanism remains to be unveiled. Furthermore, other factors apart from iron loading per se may also contribute to the observed alteration in resting and exercise torsional mechanics and require further clarification. Finally, whether the findings in the mouse model of iron overload may be translated to human subjects with beta-thalassaemia major remain uncertain.

## Conclusions

This study demonstrates impaired resting and dynamic LV torsional mechanics in patients with beta-thalassaemia major. Furthermore, we found in these patients a close relationship between LV systolic torsion and diastolic untwisting and an association between myocardial iron overload and worse diastolic untwisting. Importantly, cell and animal models of iron overload provide a novel piece of evidence for a potential role of titin degradation in the alteration of LV torsional mechanics observed in patients.

## Competing interests

The authors declare that they have no competing interests.

## Authors’ contributions

YFC has been involved in design and overall supervision of the study. SNL recruited the subjects and performed the echocardiographic studies and analyses. MPC carried out all the experiments. SNL and MPC contributed equally to the writing of the manuscript. WWL and YCH performed the cardiac magnetic resonance and interpreted the data. SY Ha and GCC recruited the subjects and interpreted the data. All of the authors critically revised the manuscript and have read and agree to the manuscript as written.

## Pre-publication history

The pre-publication history for this paper can be accessed here:

http://www.biomedcentral.com/1471-2261/14/49/prepub

## Supplementary Material

Additional file 1 Supplementary methods. Click here for file
