# Supplementary material for: Left ventricular torsional mechanics and myocardial iron load in beta-thalassaemia major: a potential role of titin degradation
Source: BMC Cardiovasc Disord. 2014 Apr 12;14:49. doi: 10.1186/1471-2261-14-49 (PMC4021258; doi:10.1186/1471-2261-14-49)
Supplement: Additional file 1 — Supplementary methods. [file 1471-2261-14-49-S1.doc]

**Additional file 1**

**Data Supplement**

**Methods**

**MRI assessment of iron load in patients**

An eight-channel cardiac array coil was used to image a single 10 mm mid-ventricular short axis slice at 8 echo times with ECG gating in a single breath-hold. The blood signal was suppressed by double inversion recovery pulses. Data were acquired every other cardiac cycle. For T2^*^ measurement, the region of interest selected was the full thickness of LV septum. For each of the images, the signal intensity of the region of interest was measured using commercial software (CMR tools, Imperial College, London, UK). The signal intensity data was plotted against echo times to derive a decay curve and T2^*^ measurements were derived accordingly by the formula y=Ke-TE/T2^*^, where y is image signal intensity, Ke a constant, and TE echo time.

**Culture of HL-1 cardiomyocytes**

Cells were grown in culture vessels pre-coated with 0.02% gelatin (Difco, Fisher Scientific, Suwanee, GA, USA) - 5 μg/ml fibronectin (Sigma, St Louis, MO, USA) solution at 37°C in a humidified 5% CO_2_ incubator, maintained in Claycomb Medium (SAFC Biosciences, Sigma, St Louis, MO, USA) supplemented with 10% fetal bovine serum (Sigma, St Louis, MO, USA), 0.1 mM norepinephrine (Sigma, St Louis, MO, USA), 2 mM L-glutamine (Invitrogen, Life Technologies, Grand Island, NY, USA) and penicillin/streptomycin (100 U/ml:100 μg/ml) (Invitrogen, Life Technologies, Grand Island, NY, USA). The medium was changed approximately 5 days per week.

**Titin gel electrophoresis**

Heart tissue from B6D2F1 mice was homogenized on ice in lysis buffer (100 mM NaCl, 1 mM EDTA, 1% Triton X-100, 10% Glycerol, 0.1% SDS, 0.5% Sodium deoxycholate, 10 mM Tris-HCl, pH 7.4) for protein extraction within 5 min. Protein concentration was determined by BCA Protein Assay Kit (Thermo Fisher Scientific, Rockford, IL USA). Equal amounts of sample (80 μg) were supplemented with protease inhibitor accordingly (Roche Applied Science, Indianapolis, IN, USA), as well as sample buffer (8 M urea, 2 M thiourea, 3% SDS, 75 mM DTT, 0.025% bromphenol blue, and 50 mM Tris-HCl, pH 6.8) in 4 times of the lysate volume. The mixture was vortexed thoroughly and heated at 60 ºC for 10 min, followed by another vortex and subsequent centrifugation for 5 min at 13200 g. Supernatant was collected for gel electrophoresis. For the electrophoresis of 2% polyacrylamide slab gel strengthened with 0.5% agarose, a 15% gel plug was made at the bottom of the gel sandwich. The final concentration of the gel plug was composed of 10% gel buffer (1% SDS, 0.2 M Sodium acetate, 20 mM EDTA, 0.4 M Tris, pH 7.4 with acetic acid), 15% acrylamide (acrylamide:N,N’-methylenebisacrylamide = 20:1), 0.05% ammonium persulfate (APS), and 0.1% tetramethylethylenediamine (TEMED). The gel plug was left to polymerize for about 1 hour, with 0.5-1 cm high at the bottom. The resolving gel was made of agarose solution and acrylamide solution. 1.5% SeaKem LE agarose (Lonza, Rockland, ME, USA) was heated to dissolved and kept at 45-60 ºC. Acrylamide solution was prepared with 15% gel buffer, 15% glycerol, 3% acrylamide (20:1), and 0.011% APS. The acrylamide solution together with gel cassette and pipette were warmed to 40-60 ºC. Warm agarose solution was added in to the final concentration of 0.5%. 0.075% TEMED was added. Gel polymerization was performed immediately in ice bath for the first 5 min followed by one more hour at room temperature.

For electrophoresis, the comb was carefully removed from the gel cassette, and the electrode chamber was set with 1:10 diluted gel buffer, with 7.5 μM β-mercaptoethanol addition to the upper chamber. After sample loading, electrophoresis was performed at 7.5 mA per gel plate for 30 min and then 15 mA per plate in succession until bromphenol blue (marker dye) reached the upper border of the gel plug. It required about 4.5 hours. After the run, the gel was stained with 0.1% coomassie brilliant blue R-250 in 50% methanol and 10% acetic acid over night at room temperature with agitation. Stained gel was subsequently destained with several changes of 50% methanol and 10% acetic acid and then once with 5% methanol and 7% acetic acid, to clear the background. Stained protein bands were scanned and analyzed with Quantity One 1-D Analysis Software (Bio-Rad, Hercules, CA, USA).
